# Effect of Sodium Dodecyl Sulfate Adsorption on the Behavior of Water inside Single Walled Carbon Nanotubes with Dissipative Particle Dynamics Simulation

**DOI:** 10.3390/molecules21040500

**Published:** 2016-04-15

**Authors:** Minh D. Vo, Dimitrios V. Papavassiliou

**Affiliations:** School of Chemical, Biological, and Materials Engineering, University of Oklahoma, Norman, OK 73019-1004, USA; minhvo@ou.edu

**Keywords:** carbon nanotubes, confined water, adsorption, CNT suspensions, SDS, dissipative particle dynamics, suspension stability

## Abstract

Dissipative particle dynamics (DPD) simulations were utilized to investigate the ability of sodium dodecyl sulfate (SDS) to adsorb inside a single-walled, arm-chair carbon nanotube (SWCNT), as well as the effect of surfactant on the properties of water inside the SWCNT. The diameter of the SWCNT varied from 1 to 5 nm. The radial and axial density profiles of water inside the SWCNTs were computed and compared with published molecular dynamics results. The average residence time and diffusivity were also calculated to show the size effect on mobility of water inside the SWCNT. It was found that nanotubes with diameter smaller than 3 nm do not allow SDS molecules to enter the SWCNT space. For larger SWCNT diameter, SDS adsorbed inside and outside the nanotube. When SDS was adsorbed in the hollow part of the SWCNT, the behavior of water inside the nanotube was found to be significantly changed. Both radial and axial density profiles of water inside the SWCNT fluctuated strongly and were different from those in bulk phase. In addition, SDS molecules increased the retention of water beads inside SWCNT (d ≥ 3nm) while water diffusivity was decreased.

## 1. Introduction

Carbon nanotubes (CNTs) have received a lot of attention both in fundamental studies and applications, because of their special thermal, mechanical, and electrical properties [1,2]. They are allotropes of carbon in a cylindrical nanostructure with a length-to-diameter ratio that can be significantly large (up to millions) [3]. Basically, CNTs can be visualized as rolled up graphene layers forming a tubular structure. The hollow part of the structure, inside the CNTs, could be considered as a confinement space where certain compounds can enter [4]. Therefore, there are interesting possible applications for open-ended CNTs, such as gas adsorbents [5,6], using them as molds to form one dimensional (quantum) systems [7,8], place catalysts on them [9], or use them as molecular filters for water treatment [10]. Hence, the adsorption and diffusion of chemical species (like water, gases *etc.*) inside CNTs should be well-understood to design and support such applications. 

The adsorption of chemical compounds inside CNTs has been verified with both experiments and simulations. Davis *et al.* used the internal surface of CNTs to immobilize proteins and enzymes [11]. It was revealed that CNTs could act as a benign host with an ability to encapsulate protein molecules within a protected environment. Fujiwara and co-workers investigated the adsorption of nitrogen and oxygen gases inside and outside single-walled CNTs (SWCNTs) by obtaining adsorption isotherms and X-ray diffraction studies [12]. It was found that the hollow space inside the nanotubes exhibited stronger attraction than the interstitial channels created when CNTs formed bundles. Pan *et al.* showed the striking enhancement of producing ethanol from CO and H_2_ by using Rh particles confined inside CNTs [13]. They reported experimental findings that the overall formation rate of ethanol inside CNTs was higher than that on the outside, even though the latter was much more accessible to CO and H_2_. With respect to simulation studies, the flow of water through CNTs was investigated with molecular dynamics (MD) simulations [14,15,16]. It was found that the hydrophobic surface of the CNT interior gives a significant enhancement for water flow through it. The nano-sorption and mobility of water, tyrosol, vanilic acid, and p-coumaric acid inside smooth SWCNTs have been calculated with MD simulations [17]. Additionally, Arai *et al.* studied the self-assembly and polymorphic transition of surfactant in water within a nanotube (inner diameter of around 8.4 nm) by using dissipative particle dynamics (DPD) simulation [18]. They presented evidence of a cornucopia of polymorphic structures of surfactant assemblies on the inner nanotube surface.

In this manuscript, we quantify the change of water behavior inside SWCNT in the presence of surfactant adsorption, and the ability of SDS to diffuse into SWCNTs of different size (inner diameter between 1 and 5 nm). The transport properties of water, such as diffusivity, residence time, and radial and axial density distribution profiles, with and without surfactant adsorption were examined. This study provides insights into the dynamics and morphology of water and surfactants in nano-confined structures.

## 2. Results and Discussion

### 2.1. The Diffusion of Water inside SWCNTs of Different Diameters

**Figure 1:**
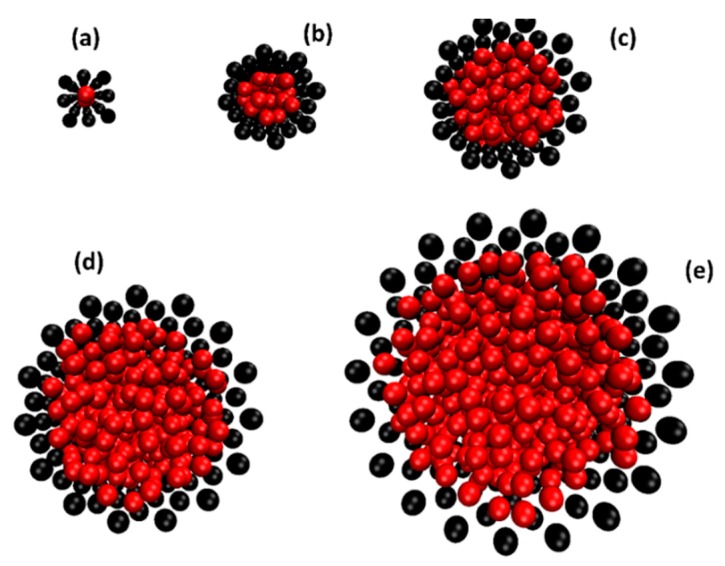
A snapshot at equilibrium (top view) of water beads inside an (8, 8) (**a**); (15, 15) (**b**); (22, 22) (**c**); (30, 30) (**d**); and a (37, 37) (**e**) SWCNT at 298 K. The CNT and water are shown in black and red beads, respectively.

In this section, the distribution of water inside different diameters (d) of open-ended SWCNTs was investigated at room temperature. The chosen SWCNTs had armchair chirality and diameters of 1, 2, 3, 4 and 5 nm, corresponding to chirality indexes of (8, 8), (15, 15), (22, 22), (30, 30), and (37, 37), respectively. During the simulation runs, the SWCNTs were kept stationary at the center of the simulation box (box dimensions: 11.49 × 11.49 × 30.64 nm^3^). All SWCNTs tested had the same length of 20 nm, irrespective of diameter. In Figure 1 we show the density distribution of water beads inside different SWCNTs after the system reached equilibrium. Water beads were able to go into the hollow space in all cases of open ended SWCNTs in our simulation. In DPD simulations, the behavior of beads containing more than one molecule, or containing clusters of atoms within a molecule, like a monomer for polymer simulations, is simulated rather than the behavior of individual atoms.

**Figure 2:**
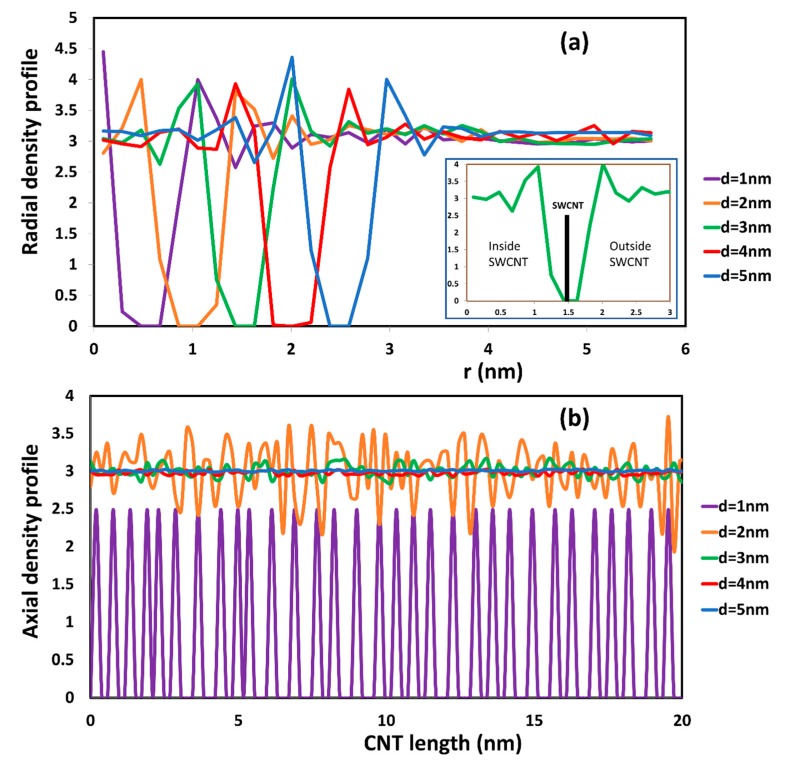
Radial (**a**) and axial (**b**) density profile of water inside different SWCNT in the equilibrium state. The inset plot in Figure 2a is an enlargement of the radial density profile with respect to distance (r) from 0 to 3 nm in the case of the SWCNT with diameter of 3 nm. The thick black line designates the position of the CNT wall. It is seen that the density profile to the right (exterior of the CNT) and to the left (interior of the CNT) is the same.

In order to evaluate accurately the order of water inside each SWCNT, the radial and axial distribution profile of water inside different SWCNTs are presented in Figure 2. In our calculation, the number density of water was selected to be 3. For SWCNT (8, 8), it is seen that the water was ordered in a single-file chain (see Figure 1a and the purple line in Figure 2b). Individual water beads were arranged along the length of SWCNT with d = 1 nm. The axial distribution in Figure 2b indicates clearly the difference of water inside the SWCNTs and in the bulk phase. The center of the nanotube is at r = 0 in Figure 2a, and the nanotube wall is at r = 0.5 nm, 1 nm, 1.5 nm, 2.0 nm, and 2.5 nm, depending on the SWCNT. For the d = 1 nm tube, there is a single density peak inside the nanotube and two distinct peaks outside the CNT. 

Along the length of the SWCNT, the axial density profile of water for the d = 1 nm nanotube is always below the density of water in the bulk phase, and it vibrates strongly from 0 to 2.5. The spacing between density peaks is rather even, and the peaks are periodic with periodicity of about 0.67 nm. This is an indication of the arrangement in a single chain of the water, as is also seen in Figure 1a. In MD simulations, it has been shown that the configuration of water in SWCNTs of chirality (8, 8) was in staked pentagons [17]. In the present case, each water DPD bead represents five water molecules, instead of individual molecules, like in the MD simulation. However, the radial and axial density profiles from DPD and MD simulations are very similar.

For SWCNTs with d ≥ 2 nm (see Figure 1b–e), the configuration of water beads inside the SWCNT is similar to that in the bulk phase. This observation is confirmed by the radial and axial distributions seen in Figure 2. For the larger diameter SWCNTs, as seen for example in the inset of Figure 2a for a SWCNT with d = 3 nm, the density profiles towards the inner and towards the outer side of the CNT wall appear to be almost symmetric. In MD simulation, it has been observed that the structure of water inside a SWCNT with d > 2 nm is bulk-like liquid behavior [19]. In the region close to the CNT surface, there is a peak of radial density that is higher than bulk density. This peak has often occurred in MD simulations for water molecules [20]. Beyond that point, the radial density oscillated around the bulk density both inside and outside of the CNTs. Along the length of the CNT, the density of water fluctuated around the bulk density of water. In other words, the density profile of water inside a CNT with d > 2 nm is the same as to that outside the CNT wall towards the bulk phase. These results and agreement with the MD results provide strong evidence that the mesoscopic DPD simulation can be used to study the transport of water inside confinement (like the interior of a CNT).

Other transport properties, like the diffusivity and residence time, can be used to characterize the behavior of water at the interior of the CNT structure. The mean squared displacement with respect to time was computed, and the slope of the line was used to determine the diffusivity of water beads inside the CNTs. The diffusion of water inside a CNT is almost the translation of water along the length of the CNT (z direction in our simulation system). Hence, the diffusion inside a CNT can be viewed as 1-D diffusion. In Table 1, we give the average residence time and the diffusivity of water inside CNTs at different diameters. Note that the water diffusivity from our calculations is the average value for all water inside the CNT. It has been observed that the water diffusivity relies on its off-center distance [21]. It makes intuitive sense that inside the CNT, the diffusion of water beads close to the CNT surface is higher than in the center of the CNT. This is caused by the hydrophobic character of the CNTs. In order to give the general diffusion data, we take average of mean square displacement of all water beads inside CNT when calculating the diffusivity.

In our DPD calculations, the diffusivity of water in the bulk was calculated to be around 3.72 × 10^−5^ cm^2^/s. This value is higher than the values for experimental diffusivity reported for water (2.43 × 10^−5^ cm^2^/s). The reason for calculating higher water self-diffusivity arises from the fact that each DPD bead represents a group of several water molecules, instead of a single molecule [22]. Admittedly, our results might not give the exact numerical value of the diffusivity, but they can give a comparative diffusion of water inside the CNT and in the bulk phase (see fourth column or results on Table 1). The diffusivity of water inside the CNT is about 34% of the bulk diffusivity for the (8, 8) SWCNT, then it increases to 68% of the bulk for the (15, 15) SWCNT and shows a maximum of being 105% of the bulk for the (22, 22) SWCNT. Then, for larger diameter SWCNTs it tends to go closer to the bulk diffusivity. These are results in agreement with results from MD simulations [21] (see fifth column or results on Table 1), where it was shown that the (20, 20) SWCNT exhibits the higher self-diffusivity of water inside the SWCNT and the (9, 9) the lower. The reason for the increase in diffusivity at higher diameters is that the hydrophobic properties of the CNT surface enhance the diffusion of water. Water is allowed to slip in adjacent regions close to the CNT surface. However, the diffusivity is also affected by the size of the confined space. It is more difficult for water to diffuse when the pore size is too narrow. Hence, the diffusivity of water inside SWCNT with diameter of 1 and 2 nm is smaller than that in the bulk phase. In terms of residence time, water took a longer time to pass through SWCNTs when the diameter of the SWCNT was reduced. Note that all SWCNTs have the same length (20 nm).

### 2.2. Can the SDS Molecules Enter the SWCNT?

In experiments, SDS has been used as a surfactant that can stabilize CNTs in an aqueous solution [23]. To determine the size of SWCNT that might allow SDS molecules to migrate and adsorb to the hollow interior of the nanotubes, simulations that included water, CNT, and SDS was performed with DPD methods. The total concentration of SDS in the system varied from 1 to 3 wt %. The simulation domain was 22.98 × 22.98 × 30.64 nm^3^ with periodic boundary conditions in all three directions.

**Figure 3:**
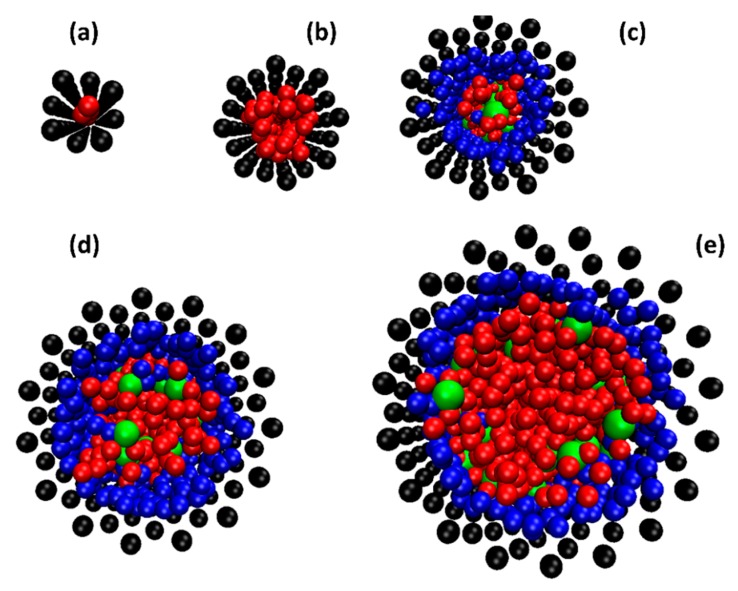
The equilibrium snapshot (top view) of SDS and water beads inside an (8, 8) (**a**); a (15, 15) (**b**); a (22, 22) (**c**); a (30, 30) (**d**); and a (37, 37) (**e**) SWCNT at 298K with total SDS concentration of 3 wt %. For SDS molecules, head and tail groups are shown in green and blue beads. CNT and water are in black and red beads, respectively. Note that the surfactant adsorption outside the CNT is removed for clarity.

**Figure 4:**
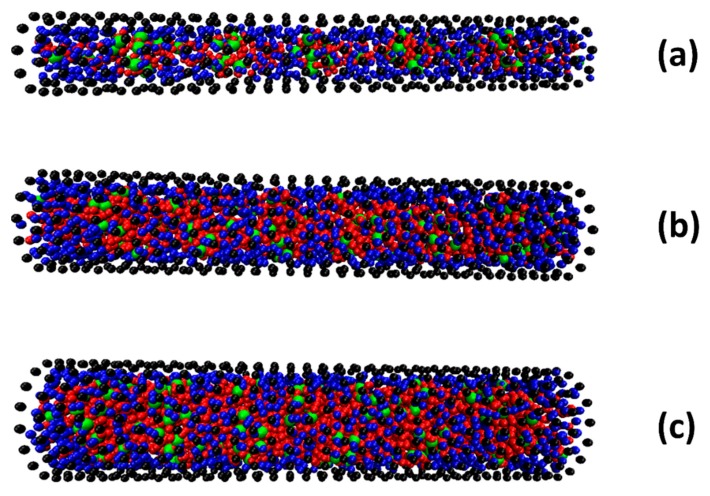
The side view of equilibrium snapshot of surfactant and water inside a (22, 22) (**a**); a (30, 30) (**b**); and a (37, 37) (**c**) SWCNT. The surfactant adsorption outside the CNT is removed for clarity. Color code is the same as that in Figure 3.

In Figure 3, we show the adsorption of SDS in different sized SWCNTs at equilibrium. The simulations showed that the SDS molecules cannot go inside the CNT if the diameter of CNT is equal or less than 2 nm. The driving force for surfactant adsorption inside the CNT is based on the attraction of the hydrophobic tail group and the CNT surface. The character of both the inner and outer surface of the CNT became more hydrophilic after the adsorption of the SDS took place. At the interior CNT surface, tails of surfactant self-assembled near to this surface and formed a monolayer. The orientation of SDS head groups were toward to the center of the CNTs. We present in Figure 4 the side view of surfactant adsorption inside the SWCNTs. Differently than the exterior surface, surfactants inside the SWCNTs can only form a random and hemi-micelle type of adsorption due to space limitations. Hemi-micellar adsorption has been observed on the exterior of multi-walled CNTs with nonionic and anionic surfactants in aqueous solutions with DPD and MD techniques [24,25,26]. Blue beads in Figure 4 indicate the tail groups of the surfactant adsorbed inside the SWCNTs. It was also seen that once the surfactant was adsorbed inside the SWCNT, it was unable to desorb from surface. Only water beads can enter and pass through the SWCNT.

The ability of SDS to enter the SWCNT is affected by the competition with water beads and by its molecular size. In the SDS-water system, surfactant molecules have to compete with water beads that can easily enter the SWCNTs. For a single molecule, the size of SDS is larger than water. Additionally, the hydrophilic heads of surfactant attract water beads (hydration process). This leads to a further increase of the size of SDS molecules. Therefore, water beads can enter the narrow space of a SWCNT easier. Another factor that needs to be considered is that the surfactant prefers to form a micelle rather than remain as a free surfactant molecule in solution. In all cases in this report, the total concentration of surfactant is higher than its CMC, meaning that both micelles and free surfactants are present in the simulation domain. In our study (the diameter of SWCNTs is less than 5 nm), we observed that only free surfactant molecules can enter and adsorb in the interior of the SWCNT surface. For SDS micelles, their outside is covered by the hydrophilic head groups of the surfactants. These micelles are more difficult to adsorb on the CNT surface due to repulsion forces (hydrophilic head groups and hydrophobic CNT surface). Therefore, SDS micelles favor propagating into the bulk water phase. For low SDS concentration (smaller than CMC), the surfactant only adsorbs on the exterior surface of the CNT where the adsorption can take place more easily and favorably. 

**Figure 5:**
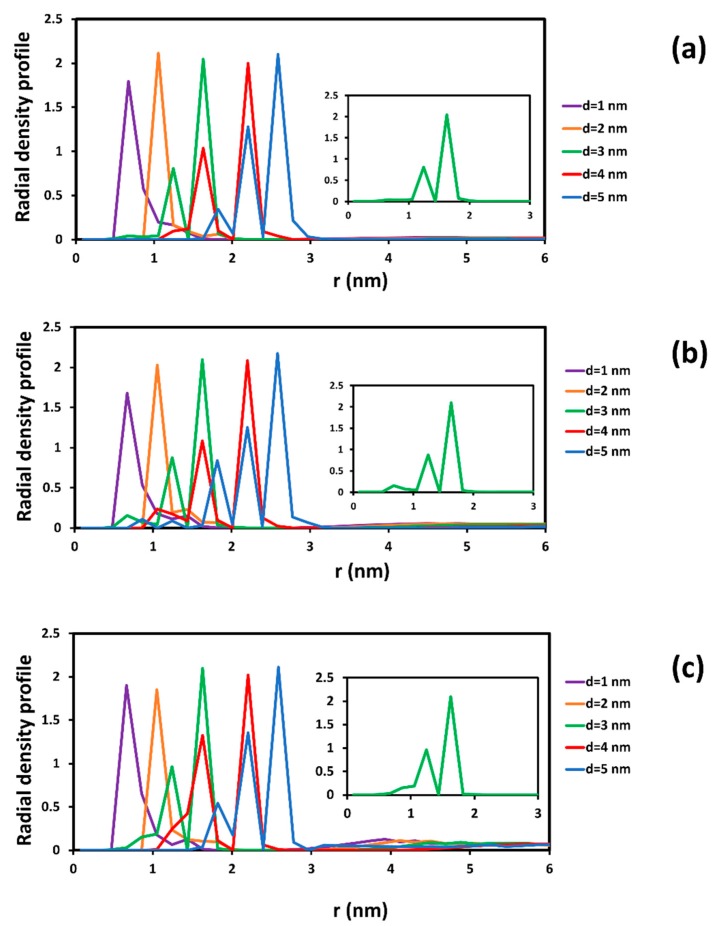
Radial density profile of surfactant inside CNTs at different total concentration of SDS: 1 wt % (**a**); 2 wt % (**b**); and 3 wt % (**c**). The insets in each figure exhibit enlargements of radial density profile with respect to distance (r) from 0 to 3 nm in case of SWCNT with diameter of 3 nm.

The radial density profile of surfactant at different total SDS concentration was also calculated in Figure 5. It indicated that the SDS surfactant prefers to adsorb on the CNT. The density of the surfactant adjacent to the CNT surface is less than the water density. In addition, the density of the surfactant in the bulk phase is significantly lower than the density of water because of the small concentration of surfactant used in this study.

The concentration of adsorbed SDS surfactant on SWCNT was computed and is presented in the fifth column of Table 2. For the same total surfactant concentration in the solution, the amount of adsorbed SDS increased with an increasing diameter of SWCNT, since the total surface area available for adsorption increased (the higher the diameter of the SWCNT, the more available area for adsorption). For the same SWCNT size, the adsorption of surfactant also increased when the concentration of SDS in the bulk increased. In addition, the adsorption could take place both on the exterior and the interior surface of SWCNT. The distribution of adsorbed surfactant between these two regions was also calculated, and is presented in the third and fourth column of Table 2. It is found that the interior surface can contribute from 1% to 13% of the total adsorption of the surfactant, depending on the size of the CNT. As already discussed, adsorption only occurred in the exterior surface of CNTs with diameters equal or smaller than 2 nm.

### 2.3. The Effect of SDS Adsorption on Water Distribution and Diffusion Inside the SWCNTs

**Figure 6:**
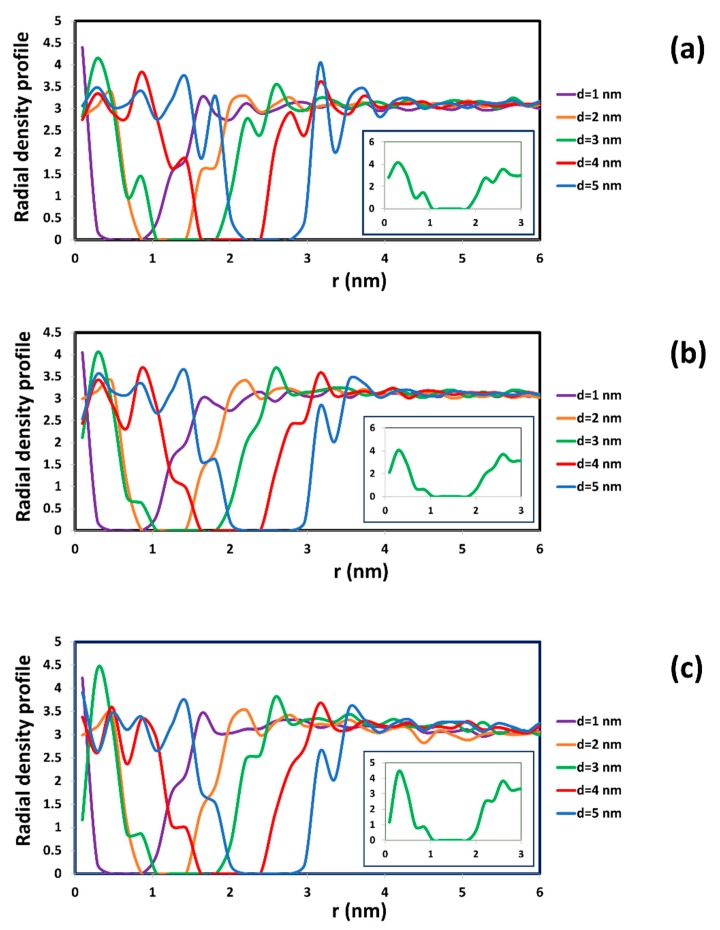
Radial density profile of water inside CNT with SDS adsorption at different total concentration of SDS: 1 wt % (**a**); 2 wt % (**b**); and 3 wt % (**c**). The insets in each figure exhibit enlargements of radial density profile with respect to distance (r) from 0 to 3 nm in case of SWCNT with diameter of 3 nm. It is seen that, contrary to the observations in Figure 2a, the radial density profile is different to the exterior and the interior of the CNT wall at r = 1.5 nm.

Obviously, the presence of adsorbed SDS remarkably affects the distribution of water inside the SWCNTs. In Figure 6, we show the radial density profile of water inside CNTs with SDS adsorption. This figure is to be compared to Figure 2 for water in a water-CNT suspension. Without SDS adsorption on the inner surface, the radial density profile of water inside the SWCNTs with diameters of 1 and 2 nm in Figure 6 is very similar to the case seen in Figure 2a. For larger diameters (d > 3 nm), water beads in the region close to the CNT shifted farther from the CNT wall, because this space was occupied by adsorbed surfactant. This leads to the expansion of the radial density profile from the surface of the CNT. In addition, the magnitude of the peak of the radial density was reduced when the concentration of SDS increased. The density of water near to the CNT surface was dependent on the amount of surfactant adsorption. It dropped when the presence of adsorbed SDS was denser on the CNT surface.

**Figure 7:**
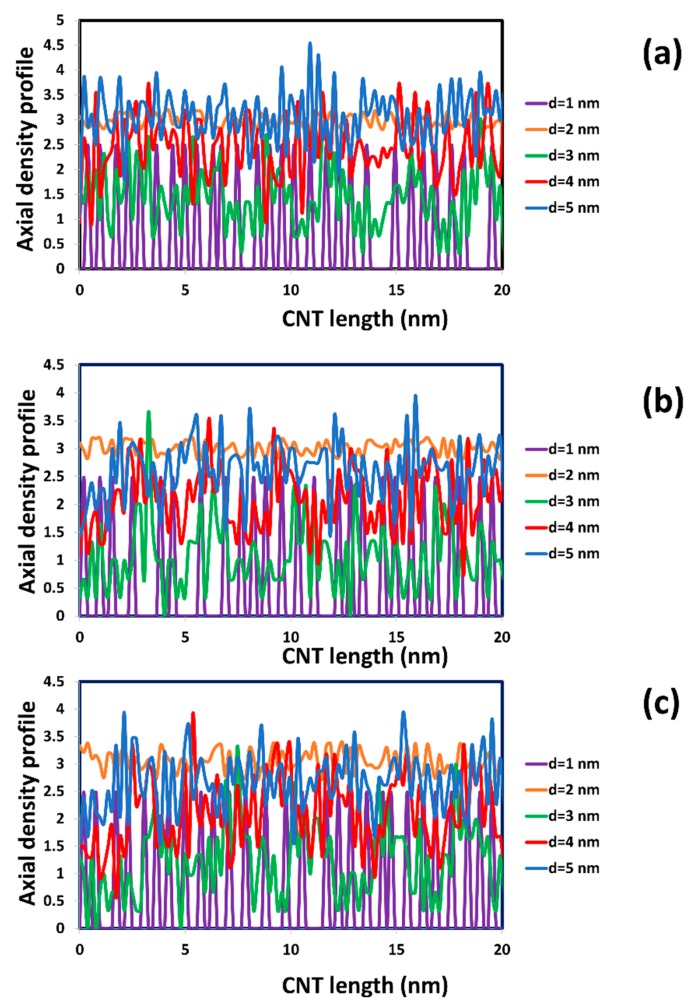
Axial density profile of water inside CNT with SDS adsorption at different total concentration of SDS: 1 wt % (**a**); 2 wt % (**b**); and 3 wt % (**c**).

In addition, the axial density profile of water also contributes to the description of the influence of the SDS surfactant on the diffusion of water through the SWCNT (see Figure 7 and compare to Figure 2b). It is found that there is a strong fluctuation of axial density along the length of SWCNTs with diameters of 3, 4, and 5 nm. It can be concluded that the surfactant adsorption changed the structure of the SWCNT interior. The distribution of surfactant inside the CNT was not uniform along the CNT length. This made the fluctuation of axial density profile to be difficult to predict and not periodic. The fluctuations are more apparent when the concentration of SDS increased. For the (8, 8) nanotube with d = 1 nm, it is notable that the peaks of water density are somewhat fewer than in Figure 2b, but the periodicity of the density peaks is not as regular as it was in Figure 2b. See, for example, in Figure 7a that there is a larger spacing than 0.67 nm between peaks at z = 14 nm and at z = 18 nm. Same is observed in Figure 6b,c at other z locations along the SWCNT axis.

The surfactant adsorption appears to have had a significant impact on diffusivity and on average residence time of water inside the CNT. In Table 3, we provide the average residence time for water and the diffusivity for the case of CNTs in the SDS adsorption. There is no effect on residence times for SWCNTs with a diameter of 1 and 2 nm. The adsorption only occurred on the exterior surface of the nanotubes. However, when the surfactant can enter the SWCNT, there is an increase of the average residence time of water. The adsorption of surfactant molecules changed the surface properties of the interior of the CNT, giving it a more hydrophilic character. The space inside the CNT also narrowed down, because some of it was occupied by surfactants. In addition, the attraction to the surfactant head groups that pointed toward the center of the nanotube made the water retention time longer. Water beads had to spend more time in order to pass through the SWCNTs in the presence of adsorbed surfactants. At the same time, the increase of average residence times led to a decline for the diffusivity of water. This decline is very obvious for SWCNTs with diameters larger or equal to 3 nm. 

For the (15, 15) SWCNT that has a diameter of 2 nm, the differences in diffusivity between the cases with and without surfactant are negligible. However, for the smallest nanotube examined, the (8, 8) SWCNT, there was an increase in diffusivity. This means that even though the average residence time of water inside the CNT is comparable, the distribution of this time is wider for the case with SDS adsorbed at the exterior of the CNT. As was noted when the water density profile along the axial direction of the nanotube was discussed (see discussion above for Figure 6 in comparison to Figure 2b), the distance between peaks in water density is not uniform for water traveling inside narrow tubes with SDS adsorbed around it. The hydrophobic groups of the SDS are adsorbed at the exterior surface of the CNT and their collective effects appear to be felt by the water inside the narrow CNT increasing the diffusivity from ~128% to 142%, as seen in Table 3.

**Figure 8:**
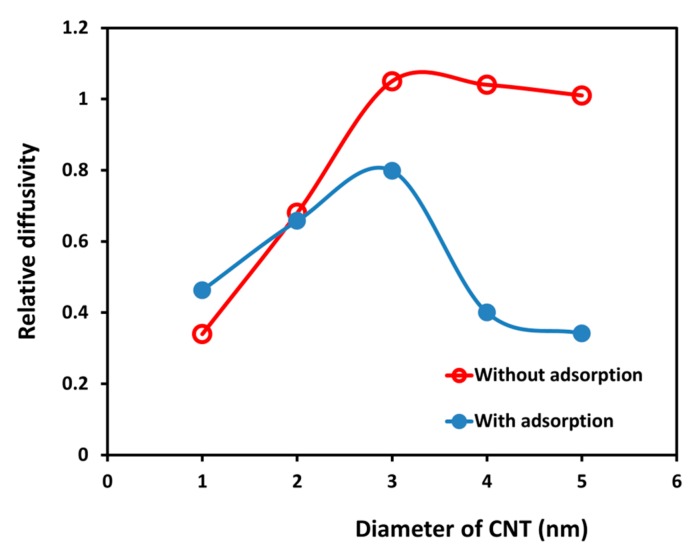
The relative diffusivity of water inside CNTs with different diameter with and without SDS adsorption. Relative diffusivity is the ratio of diffusivity of water inside the CNT to that in the bulk phase. The line in this plot is only used to connect the data and guide the eye to see the trend of changing relative diffusivity.

The effect of the SDS adsorption on the diffusivity of water inside the CNT is emphasized in Figure 8, where we present the changes of relative diffusivity with respect to the CNT diameter as the adsorption of SDS occurs. Note that relative diffusivity with surfactant adsorption is the average value of runs with different total SDS concentration, listed on Table 3. Without the presence of surfactant, the diffusivity of water inside the CNT increases with increasing CNT diameter (from 1 to 3 nm). After that, the diffusivity of water is nearly the same as the diffusivity in the bulk phase. In this case, the properties of water inside the CNT become similar to those in the bulk phase. In the occurrence of SDS adsorption, the attraction of hydrophilic head groups to water beads leads to increasing the retention of water beads inside the CNT. For small CNT diameters (d ≤ 2 nm), when the SDS cannot enter the CNT, the two cases almost coincide. However, for larger CNT diameters (d ≥ 3 nm), when the SDS can enter the CNT, the diffusivity of water is decreasing as the CNT diameter increases. In contrast to the case of no surfactant present, it does not seem that the diffusivity of water inside the CNT can be equal to that in the bulk for any CNT diameter studied, because of the attraction of hydrophilic tails of adsorbed surfactant and water beads.

## 3. Computational Details

The DPD is considered to be a coarse-grained approach to molecular level computations. It is a particle-based method utilized to determine the evolution of a many body system controlled by Newton’s equation of motion [22]:

(1) $$\frac{dr_{i}}{\text{dt}}=v_{i}$$

(2) $$m_{i}\frac{dv_{i}}{\text{dt}}=f_{i}$$

where **v**_i,_ **r**_i_, and m_i_ are the velocity, position, and mass of a particle i, **f**_i_ is the inter-particle force on particle i by all of the other particles, and t is time. Basically, this interaction force **f**_i_ is the sum of three forces, *i.e.*, the conservative force (**F**^C^_ij_), the drag or dissipative force (**F**^D^_ij_) and the random force (**F**^R^_ij_) exerted because of the presence of particles j on each particle i [22]:

(3) $$f_{i}=\underset{j\neq i}{\sum }\left(F_{\text{ij}}^{C}+F_{\text{ij}}^{D}+F_{\text{ij}}^{R}\right)$$

These forces are pairwise additive and have been expressed in the DPD literature [22] as follows:

(4) $$F_{\text{ij}}^{C}=\{\begin{array}{c} a_{\text{ij}}\left(1-\frac{r_{\text{ij}}}{r_{c}}\right)\hat{r_{\text{ij}}}\;(r_{\text{ij}}\;<\;r_{c})\\ 0\;\left(r_{\text{ij}}\;\geq \;r_{c}\right) \end{array}$$

(5) $$F_{\text{ij}}^{D}=-{\gamma w}^{D}\left(r_{\text{ij}}\right)\left(\hat{r_{\text{ij}}}.v_{\text{ij}}\right)\hat{r_{\text{ij}}}$$

(6) $$F_{\text{ij}}^{R}={\sigma w}^{R}{\left(r_{\text{ij}}\right)}_{\text{ij}}{\left(\Delta t\right)}^{-0.5}\hat{r_{\text{ij}}}$$

where **r**_ij_ = **r**_i_ − **r**_j_, $\hat{r_{\text{ij}}}{=r}_{\text{ij}}/$|**r**_ij_|, v_ij_ = v_i_ − v_j_, ∆t is the time step, r_c_ is the cut off radius and a_ij_ is the repulsion parameter between beads i and j; w^D^ and w^R^ are r-dependent weight functions that vanish for r > r_c_; $\xi_{\text{ij}}$ is a random variable following a Gaussian distribution with zero mean, unit variance, and is uncorrelated in time; γ is the friction coefficient, and *σ* is noise amplitude. Values of w^D^(r), w^R^(r), γ and *σ* can be chosen by applying the fluctuation-dissipation theorem [27]:

(7) $$w^{D}\left(r\right)={\left[w^{R}\left(r\right)\right]}^{2}$$

(8) $$\sigma^{2}=2{\gamma k}_{B}T$$

where k_B_ is the Boltzmann constant, and *T* is the temperature of the system. Furthermore, it has been demonstrated that DPD maintains the correct hydrodynamic properties of a system, because the forces **F**^C^_ij_, **F**^D^_ij_, and **F**^R^_ij_ conserve momentum locally [22,27].

**Figure 9:**
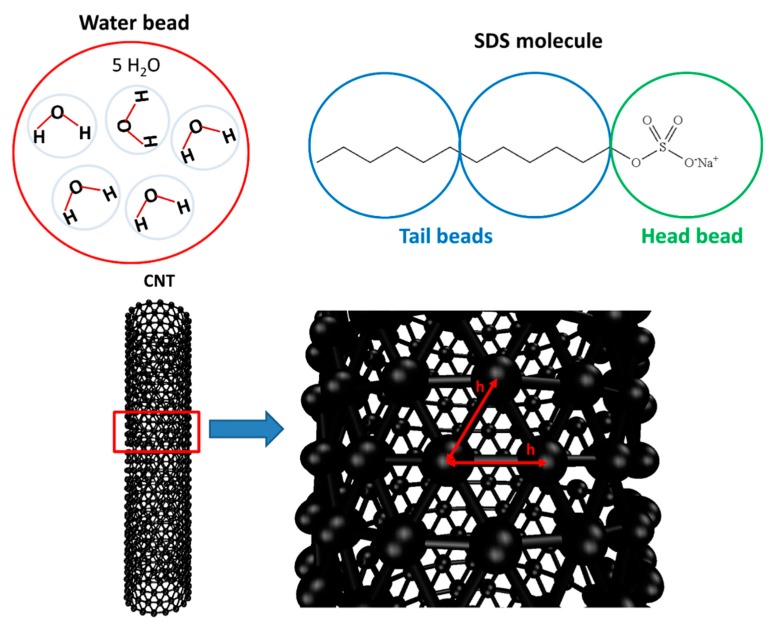
A schematic representation of the coarse-grained model for water, SDS surfactant molecule, and CNT in our DPD simulation. Each water bead represents five water molecules. The SDS molecule has three beads, including one head and two tail beads. For the CNT, the distance (h) between two nearest beads is 0.5 r_c_.

A schematic representation of the coarse-grained model used in this report is presented in Figure 9, where the model for SDS molecules, water, and the SWCNTs is presented. Five water molecules were grouped into one simulation bead. Then, the volume of a water bead (W) has to be around 150 Å^3^ [28]. The number density of water (ρ) was set to a value of 3. Thus, the length scale (r_c_) of the DPD simulation was 0.766 nm. For the surfactant molecule, its volume is about 410 Å^3^ [29]. We can then consider a single SDS molecule to be equivalent to 3 water beads: two beads are tail groups (T) and another is head group (H). Two consecutive beads of SDS molecule were also connected by harmonic bond potential with the spring constant (k_s_) equal to 100 k_B_T/r_c_^2^.

The CNT was considered as a rigid hollow cylinder in our simulation. Since the objective of our study is to study the properties of water and surfactant inside open-ended CNTs, it was not needed to have additional bonds and angular potentials among CNT beads. Additionally, Thomas and McGaughey proved that the structure of water inside the CNT is not affected by fixing the carbon atoms of CNT [19]. All CNT beads were arranged in an equilateral triangular lattice with nearest neighbor distance h = 0.5r_c_ (see Figure 9).

All DPD simulations were carried out using the open-source LAMMPS software package [30]. For simplicity, reduced units were used in DPD calculations. The temperature of the system was kept constant at reduced temperature k_B_T = 1 (equivalent to 298 K). The pressure of the system was about 0.1 MPa. The canonical ensemble (NVT − constant-temperature, constant-volume) was also applied in all DPD calculations. The noise amplitude and friction coefficients were set to σ = 3 and γ = 4.5, respectively [22]. Additionally, all the simulations were carried out for 3 × 10^6^ steps with a time step of 0.01 in reduced DPD units. The visual molecular dynamics (VMD) software was used to visualize all snapshots illustrated in this work.

**Figure 10:**
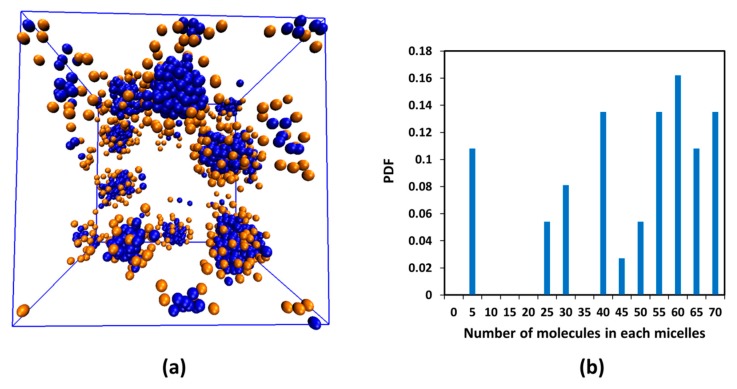
(**a**) The equilibrium snapshot of 2 wt % SDS in water. All of the water beads are removed for clarity. Blue and orange beads represent the tail and head beads of the surfactant, respectively; (**b**) the distribution of number of molecules in each SDS micelle in water.

Initially, positions of water and surfactant molecules were randomly distributed in the whole simulation domain. Once every 100 time steps, configurations of the whole system were saved for further analysis. The diffusion constant of water was used to determine the time scale in the DPD simulation [31]. The slope of the mean square displacement of water beads with time is equal to six times the water DPD diffusion constant (D_w_), according to Einstein’s diffusion theory. Thus, the time scale (τ) in our DPD simulation was 0.044 ns. With respect to the repulsion parameter, we adopted results from published reports for DPD simulations [22,24,32,33,34]. A summary of all repulsion parameters applied in this manuscript is presented in Table 4. Note that electrostatic interactions were indirectly added into the DPD algorithm by choosing the repulsion parameters of head groups—water and tail groups—water [33]. In solution, surfactant could agglomerate together to form a micelle. In each aggregate, the hydrophobic tail assembled in the center (blue beads) and the polar head beads (orange) were on the outside (see Figure 10a). Water beads have a strong attraction to head groups and repel tail groups of SDS.

The SDS concentration in our report was higher than its CMC. From experiment, it showed that CMC of SDS surfactant is around 8.1–8.4 mM (equivalent to 0.23 wt %) [35]. In our DPD calculation, CMC of surfactant was determined by counting the number of free molecules in the solution. Figure 10 showed the distribution of number of molecules in each micelle. If the number of molecules in any aggregate was lower than 5, it could consider as free surfactants. It was found that the CMC of surfactant in our DPD simulation is around 0.25 wt %. There is a good agreement between simulation and experimental data in terms of CMC.

## 4. Conclusions

Our results indicate that DPD can be used to study the transport properties of water in the confined space of SWCNTs. The properties of water in SWCNT from DPD calculation agreed well with MD simulations. For SWCNTs with d ≥ 2 nm, the radial and axial density of water inside the SWCNT is comparable with those in the bulk phase. Additionally, its diffusivity was enhanced due to the hydrophobic surface of SWCNTs. For SWCNTs with d < 2 nm, there was a marked difference in the density profile between water in the interior of the SWCNTs and in the bulk phase. The diffusivity of water was reduced in those cases, in agreement with prior MD results, because of the ordering of water molecules in almost single file within the interior space of the SWCNTs with small diameters.

It was found that SDS molecules can enter a SWCNT if its diameter is equal or greater than 3 nm. The adsorption of surfactant can occur spontaneously in both the interior and exterior surface of the SWCNT (d ≥ 3 nm). Obviously, the percentage of surfactant adsorption on the outside of a SWCNT is always dominant, because of the higher available surface for adsorption. The surfactant adsorption inside SWCNT increased with the increasing of SWCNT diameter. Depending on the concentration of surfactant, SDS inside SWCNT can accumulate from 1% to 13% of the total of adsorbed surfactant, and the adsorbed molecules self-assemble in hemi-micellar and random forms.

Finally, the adsorption of SDS inside SWCNT led to the change of water properties inside the SWCNT. For SWCNTs with d ≥ 3 nm, the radial and axial density profile of water inside SWCNT were remarkably different from those in bulk phase. Moreover, the average residence time of water inside SWCNT was increased in the occurrence of surfactant adsorption. The interior of the SWCNT surface was more hydrophilic and able to hold water longer. As a result, the diffusivity of water also decreased with increasing surfactant adsorption inside the SWCNT. In the case of the narrowest SWCNT considered in this study, the water axial diffusivity increased, because of the collective hydrophobic effects of SDS adsorbed on the exterior of the nanotube that can be felt in the confined space.

## Figures and Tables

**Table 1:** Diffusivity and average residence time of water inside SWCNT.

Diameter of CNT and Chirality	Average Residence Time (ns)	Diffusivity of Water inside SWCNT (cm^2^/s)	Diffusivity Ratio between Water inside SWCNT and in Bulk Phase (from Our DPD Simulation)	Diffusivity Ratio between Water inside SWCNT and in Bulk Phase (from MD Simulation) [21]
1 nm (8, 8)	57.1	1.27 × 10^−5^	0.34	0.21
2 nm (15, 15)	26.3	2.53 × 10^−5^	0.68	0.95
3 nm (22, 22)	18.6	3.91 × 10^−5^	1.05	1.15
4 nm (30, 30)	17.9	3.86 × 10^−5^	1.04	1.06
5 nm (37, 37)	18.3	3.77 × 10^−5^	1.01	1.02

**Table 2:** Percentage adsorption on the inside and outside surfaces of SWCNTs.

Diameter of CNT	Total Concentration of SDS (wt %)	Percent Adsorption Inside CNT	Percent Adsorption Outside CNT	Total SDS Adsorption (wt %)
1 nm	1	0	100	0.45
	2	0	100	0.44
	3	0	100	0.47
2 nm	1	0	100	0.64
	2	0	100	0.68
	3	0	100	0.67
3 nm	1	1.3	98.7	0.82
	2	1.5	98.5	1.05
	3	2.3	97.7	0.98
4 nm	1	12.9	87.1	0.88
	2	8.7	91.3	1.25
	3	10.1	89.9	1.27
5 nm	1	12.3	87.7	0.90
	2	13.3	86.7	1.48
	3	13.1	86.9	1.50

**Table 3:** Diffusivity and residence time of water in the effect of SDS adsorption.

Diameter of CNT	Total Concentration of SDS (wt %)	Average Residence Time (ns)	Diffusivity of Water Inside SWCNT (cm^2^/s)	Ratio between Diffusivity of Water Inside SWCNT with and without SDS Adsorption
1 nm	1	58.1	1.80 × 10^−5^	1.42
	2	63.0	1.61 × 10^−5^	1.28
	3	61.7	1.78 × 10^−5^	1.41
2 nm	1	25.4	2.49 × 10^−5^	0.98
	2	24.5	2.59 × 10^−5^	1.02
	3	27.7	2.29 × 10^−5^	0.90
3 nm	1	20.6	3.07 × 10^−5^	0.79
	2	20.8	3.04 × 10^−5^	0.78
	3	22.3	2.84 × 10^−5^	0.73
4 nm	1	43.8	1.45 × 10^−5^	0.37
	2	40.6	1.56 × 10^−5^	0.40
	3	42.8	1.48 × 10^−5^	0.38
5 nm	1	47.6	1.33 × 10^−5^	0.35
	2	51.7	1.22 × 10^−5^	0.32
	3	49.4	1.28 × 10^−5^	0.34

**Table 4:** All repulsion parameters for water (W), CNT, and SDS beads in k_B_T units. H and T represent head and tail beads of SDS molecule.

	W	CNT	T	H
W	25	60	80	15
CNT		25	25	40
T			15	80
H				35
